# Unrecognized Pulmonary Hypertension in Non-Cardiac Surgical Patients: At-Risk Populations, Preoperative Evaluation, Intraoperative Management and Postoperative Complications

**DOI:** 10.3390/jcdd10090403

**Published:** 2023-09-19

**Authors:** Roop K. Kaw

**Affiliations:** Department of Hospital Medicine, Cleveland Clinic, Outcomes Research Consortium, Cleveland, OH 44195, USA; kawr@ccf.org

**Keywords:** pulmonary hypertension, non-cardiac surgery, perioperative monitoring, right ventricular failure

## Abstract

Pulmonary hypertension is a well-established independent risk factor for perioperative complications after elective non-cardiac surgery. Patients undergoing cardiac surgery are routinely evaluated for the presence of pulmonary hypertension in the preoperative period. Better monitoring in the postoperative critical care setting leads to more efficient management of potential complications. Data among patients with pulmonary hypertension undergoing elective non-cardiac surgery are scant. Moreover, the condition may be unidentified at the time of surgery. Also, monitoring after non-cardiac surgery can be very limited in the PACU setting, as opposed to the critical care setting. All these factors can result in a higher postoperative complication rate and poor outcomes.

## 1. Introduction

Driven by the ever-increasing prevalence of heart disease, an aging population, and obesity, the prevalence of pulmonary hypertension (PH) is also rising [1]. Moreover, patients from all these backgrounds are also often in need of elective non-cardiac surgery (NCS), and PH may often be undiagnosed at the time of surgery. PH increases the 1-year standardized mortality rate by a factor of seven, which can further increase exponentially from the additional stresses of anesthesia, hypoxia, hypercapnia, mechanical ventilation, and sympathetic stimulation during surgery [1]. Perioperative outcome data from several small studies point to very high morbidity and mortality rates, although the exact causes of mortality are harder to establish using large databases. In addition to an increased emphasis on preoperative diagnosis, the crux of the perioperative management of PH is preserving RV function and accommodating rapid changes in preload and afterload. More recently, PAH risk assessment scores have been utilized in combination with clinical risk factors, whereas the commonly used revised cardiac risk indices (RCRIs) and the national surgical quality improvement program (NSQIP) indicators lead to an underestimation of the perioperative risk. Despite proper patient selection, considering types of anesthesia, lower-risk surgical procedures, and the center of excellence, perioperative outcomes remain poor in patients with PH.

## 2. Perioperative Impact

In the largest-yet reported series of more than 17 million hospital admissions (Healthcare Cost and Utilization Project’s National Inpatient Sample) in the United States, PH was reported present in 0.81% (1 out of every 125) of patients admitted for NCS between 2004 and 2014 [2]. Amongst all the different non-cardiac surgeries, PH has been reported to be more likely among patients needing transplantation, vascular, thoracic, and orthopedic surgeries.

### 2.1. Perioperative Complications

Mortality ranges between 0 and 18% among elective non-cardiac surgical procedures and is higher after emergency surgery. In the largest reported series, 4.4% of patients died in the hospital after surgery, and 8.3% developed major adverse cardiac events (MACEs) [2]. Perioperative complications reported after NCS include right ventricular (RV) failure, respiratory failure, myocardial infarction (MI), arrhythmias, stroke, hemodynamic instability, and longer intensive care unit length of stay (ICU LOS) [2,3,4,5]. Patients with group 1 PH are at the highest risk of complications compared with those with group 2 PH [4].

### 2.2. Occult PH

Of the 143,846 surgical hospitalizations with a diagnosis of PH in the National Inpatient Study (NIS), echocardiography data were not recorded, and right heart catheterization (RHC) was performed in 2.1% [2]. Younger patients (18–39 years) and those with pulmonary arterial hypertension (PAH) or WHO group 1 PH were more likely to get RHC (4.3%). Although RHC is still the gold standard for the diagnosis of PH, echocardiography is the main screening tool for PH, and a higher prevalence of PH has been reported by echocardiogram in the aging population [6]. Since preoperative echocardiogram is not the standard of practice, and older people are less likely to undergo RHC (0.9% if above age 80), PH can be underestimated during preoperative evaluation. Older men, obese patients, and especially those with obstructive sleep apnea (OSA) can have occult PH. In addition to OSA, increased blood volume and the resultant cardiac output with changes in VQ mechanics have been attributed to the development of PH in the morbidly obese. A 1 kg/m^2^ increase in body mass index (BMI) is associated with a 0.08 L/min increase in cardiac output and a 1.35 mL increase in stroke volume [7].

### 2.3. Pathophysiology of Perioperative Right Ventricular Failure

As the severity of PH increases, the RV is unable to adapt to and accommodate the high pressures in the pulmonary circulation. This process, called uncoupling, results in increased RV filling pressures, leading to RV dilation and failure [8]. The right atrial pressure (RAP) exceeds 15 mmHg and the cardiac output decreases, which decrease exercise capacity. This is why exercise capacity is an important determinant of the severity of PH and perioperative outcomes as much as the absolute value of the mean pulmonary arterial pressure (MPAP). Eventually, the coronary perfusion to the RV decreases, and repetitive RV ischemia leads to more uncoupling and worse RVF [9].

Several perioperative situations and interventions can cause uncoupling due to severe fluctuations in MPAP, filling pressures, and cardiac output. (Table 1) Laryngoscopy can induce sympathetic stimulation, which can increase pulmonary vascular resistance (PVR). Vasoplegia from general anesthesia induction and a sympatholytic response from spinal anesthesia both, can result in a fall in cardiac output (CO) and severe hypotension, respectively. Positive pressure ventilation can increase the MPAP and decrease preload, especially when high tidal volumes or positive end-expiratory pressure (PEEP) are used. Hypoxia, hypercapnia, and hypothermia can increase pulmonary vasoconstriction and PVR, contributing to RVF. Among the other factors that can precipitate RVF are volume overload, surgical bleeding and/or blood product transfusion, pulmonary micro-embolism, pulmonary artery clamping and cytokine storm after tourniquet release, and flushing lines infusing pulmonary vasodilators.

## 3. Preoperative Evaluation

Symptomatic PH typically presents as exertional dyspnea and reduced activities of daily living (ADLs) and can be indistinguishable from NYHA class III-IV CHF. The first step in preoperative assessment is to assess the WHO functional class and severity of PH and RV function and decide the type of surgery. In fact, the severity of PH is reflected in exercise performance, RV dysfunction, and pulmonary hemodynamics. The overall need for surgery, especially the type of surgery, is important to assess in the presence of significant RV dysfunction.

### 3.1. Physical Examination

Physical examination provides limited overall utility in establishing the presence of PH. Examination of the patient’s neck can reveal elevated jugular venous pressure (JVP); prominent *a* waves represent higher right atrial (RA) pressures, and *v* waves represent tricuspid regurgitation. Left parasternal heave in the presence of an elevated JVP (>3 cm) and pedal edema were reported to be 100% predictive of MPAP ≥ 45 mmHg [10]. The emphasis during physical examination in the preoperative setting should be on suspecting RV dysfunction and early RV failure. Large V waves, S3/S4 gallop, pedal edema, hepatomegaly, and/or ascites already suggest RV failure [11]. Signs of end organ dysfunction like hypotension and renal failure suggest severe RV failure.

### 3.2. Preoperative Planning and Investigations

Preoperative planning and risk stratification should allow 2–4 weeks before surgery [12]. (Table 2) Highest-risk patients should be referred to centers that are equipped with resources for perioperative and intraoperative care of such patients [13]. All others need an anesthesiologist trained in transesophageal echocardiography (TEE) and mechanical circulatory support management. Arterial blood gas (ABG) should be obtained to uncover hypoxia and acidosis, which can influence pulmonary vascular resistance (PVR) [14]. Preoperative PFTs are encouraged for identifying or reassessing obstructive/restrictive lung disease, while severely decreased diffusion capacity (DLCO) can indicate severe PH [15].

Transthoracic echocardiography (TTE) is the main screening tool for PH. PA > 25 mm, RV outflow Doppler (acceleration time) AT < 105 ms, and early diastolic PR velocity > 2.2 m/s suggest PH. The severity of PH on echocardiography can be assessed via the degree of RV and RA dilation, and a TV regurgitant jet velocity of 2.8 m/s. Tricuspid annular plane systolic excursion (TAPSE), max TR velocity, RV myocardial performance index (MPI) ≥ 0.75, and right ventricular systolic pressure (RVSP)/systolic blood pressure (SBP) ≥ 0.66 have been associated with postoperative complications and early postoperative mortality [3,16]. ASA physical status, WHO functional class III-IV, and a 6 min walk distance (6 MWD) < 400 m are also important predictors of outcome [17]. The role of cardiopulmonary exercise testing (CPET) in preoperative evaluation of PH patients is unclear at this time, and further data are needed. Case cohort studies have suggested poor postoperative outcomes in patients with group 3 PH with reduced VO_2_ peak and abnormal VE/VCO_2_ after non-cardiac surgery [18,19].

Right heart catheterization (RHC) is not routinely recommended before surgery but can be considered. Which category of patients may benefit is not yet clear. Although RHC can provide clarity about the hemodynamics, especially MPAP, the latter as an absolute number by itself is not sufficient to determine the severity of and the outcomes related to PH [8,20] as long as the RV adapts to the afterload. At a point in time when the RV fails, the RV filling pressures rise, and functional capacity declines. The cardiac index (CI) and NYHA are better measures of RV response to coupling (with MPAP) and therefore can be better predictors of perioperative outcomes than MPAP by itself.

Pulmonary vasodilator medications should be continued through the day of surgery to avoid severe rebound PH crisis [21]. If it is decided to use these medications prior to surgery time, a period of 6–12 weeks should be allowed for optimization.

### 3.3. Preoperative Risk Stratification

Comprehensive risk assessment, prognosis, and survival in PH depend on many diverse factors and are best achieved using composite scoring scales like REVEAL [5]. Current perioperative guidelines do not provide much objective evidence to guide preoperative evaluation of patients with PH. Therefore, some investigators have developed perioperative risk stratification strategies based on composite scores of routinely obtained patient-related variables in PH and procedural risk. One such composite score called the pulmonary hypertension perioperative risk score (PHPR) showed better discrimination and less need for further preoperative workup or hemodynamic studies but is awaiting validation [22].

## 4. Intraoperative Management

### 4.1. Anesthesia and Pulmonary Hypertension

Wherever possible, monitored anesthesia care (MAC) using local anesthesia ± peripheral nerve blocks with conscious sedation is the safest strategy. The primary concern with general anesthesia, especially with severe PH, is related to the hemodynamic effects of mechanical ventilation on the RV. In addition, induction can induce hemodynamic instability, especially when using premedications opioids and benzodiazepines together, which can predispose to hypoxia/hypercarbia and hence elevated PVR [12]. This can be mitigated via rapid sequence induction and mask ventilation with prolonged exhalation to decrease positive intrathoracic pressure [12]. Etomidate is short-acting; it does not impact PVR as much and makes a good choice for induction [23]. Repetitive doses or continuous infusion of etomidate have been found to inhibit 11beta-hydroxylase in the adrenal and increase the risk of death [23]. Recent data do not show any difference in survival with etomidate among patients with PH who underwent endotracheal intubation and mechanical ventilation [24]. Intravenous anesthesia with propofol is safe but can affect RV contractility [25]. Among the inhalation agents, nitrous oxide can increase PVR [26]. Neuroaxial anesthesia is safer to titrate than spinal anesthesia. Single-dose bolus can have severe sympatholytic effects, and vasopressors should be handy to mitigate the decrease in mean arterial pressure (MAP) [27]. A combined spinal–epidural may be better tolerated. Smooth emergence from anesthesia is important for avoiding abrupt increases in sympathetic tone and can be best mitigated by pressure support ventilation.

### 4.2. Cardiopulmonary Monitoring

The most dreaded outcomes to monitor for are RV ischemia and acute RV dysfunction. MAP should be maintained ≥60 mmHg. Therefore, diastolic blood pressure monitoring is best conducted with the help of invasive arterial blood pressure monitoring prior to induction.

It is well understood by most that central venous pressure (CVP) does not assess LV preload accurately in patients with PH. CVP should be maintained at 6–10 mmHg in most patients with stable PH/RVF. In PH patients with preserved LV function, however, CVP monitoring can help assess RA and RV filling pressures and guide volume management. A low MAP with an elevated CVP indicates a failing RV, but a falling CVP with low MAP may indicate hypovolemia. CVP can be particularly important for the detection of new-onset RHF or worsening tricuspid regurgitation (TR) with a loss of X-descent and C-wave fusion leading to a larger V wave. The additional advantages of a CVP line can include the administration of vasodilators and the measurement of central venous oxygen saturation, which can be used as a surrogate for mixed venous oxygenation.

The choice of pulmonary artery catheter (PAC) vs. transesophageal echocardiography (TEE) depends on the availability of the latter and expertise. By being able to monitor pulmonary artery systolic pressure (PASP) and RV and LV function and to guide fluid management, the use of TEE has allowed to suspect cardiac shunting, MI, or ventricular fibrillation during the intraoperative period [28]. However, early acute RV dysfunction can be missed [29]. TEE is also better at assessing biventricular function. In situations with inappropriate or exuberant use of pulmonary arterial vasodilators, it helps detect LV dysfunction resulting from high-output RV failure [12].

PAC may offer advantages in monitoring changes in PAP, mVO_2_, RV function, and cardiac output during interventions and titration of vasoactive/inotropic therapy, although the measurements may not always be reliably accurate [29,30]. PAC may not be needed during low-risk procedures in patients with mild-to-moderate PH. Significant TR and intra-cardiac shunting can hamper the accurate measurement of thermodilution CO. PAC placement can also induce arrhythmias and, rarely, PA branch rupture.

### 4.3. Airway Management

In general, hypoxia, hypercarbia, acidosis, and atelectasis should be avoided with close monitoring, as these can affect PVR and RV function. Mild hypocarbia (30–35 mmHg) is favored via adjusting the respiratory rate with continuous blood gas monitoring [31,32]. Plateau pressures are best targeted at <27 cm H_2_O by keeping tidal volumes of 6–8 mL/Kg of the ideal body weight with a PEEP < 5–10 cm H_2_O. Extreme lung volumes (both high or low), high inspiratory pressures, and PEEP can decrease preload and worsen PVR and RV function and should be avoided [32]. Therefore, a higher fraction of inspired O_2_ (FiO_2_) is preferred over PEEP after alveolar recruitment for minimizing atelectasis.

### 4.4. Medication Management

Oral prostanoids should be continued preoperatively in the form of perioperative inhaled or intravenous prostanoids, as well as when needed intraoperatively to optimize CVP and decrease RV afterload [33]. Prostanoids and endothelial receptor antagonists (ERA) should, however, be avoided in group 2 PH, as they can worsen left ventricular failure. In these situations, the focus should be on the management of underlying heart disease and diuresis [34], although inhaled nitric oxide (NO) can be given in selected patients as long as the pulmonary capillary wedge pressure (PCWP) is optimized. Systemic vasodilators like nitroprusside, nitroglycerine, or nesiritide can also help reduce PVR and increase cardiac output in group 2 PH [14]. Epoprostenol and NO have short half-lives and are most suited for use in the perioperative period. These are best used in hemodynamically unstable patients with RV failure that is unresponsive to vasopressors. Intravenous infusions and chronic oral therapies for PAH should not be abruptly interrupted (group 1 and group 4 PH). Nebulized/inhaled agents can quickly reduce RV afterload and have the additional benefit of decreasing V/Q mismatch [35]. Perioperative bridging anticoagulation should be continued in group 4 PH and is not required in group 1 PH. Calcium channel blockers, diuretics, and beta-blockers should also be continued to maintain normal sinus rhythm, except in overt RV dysfunction.

Pregnancy is best avoided in patients with known PH, but PH may be diagnosed for the first time during pregnancy. Because of the higher propensity for RVF in the postpartum period, obstetric care should be provided at a facility that has the capability to manage PH. IV prostacyclins are safe, but endothelin receptor antagonists are contraindicated.

### 4.5. Pulmonary Hypertensive Crisis

Several events/interventions during the intraoperative course can lead to sharp increases in PVR and MPAP, causing decreases in CO, hypotension, and cardiovascular collapse [34]. The PAC-guided approach to hemodynamics can identify or rule out such causes as vasodilation from induction of anesthesia, positive pressure ventilation, supraventricular arrhythmias, micro-thromboembolic PE, extubation, etc. Proper attention to induction, tidal volume, PEEP, intravascular volume status, avoiding air bubbles, or flushing lines infusing pulmonary vasodilators are some examples of practices that help prevent sudden sharp increases in PVR.

Systemic hypotension with stable CO indicates that the patient has reduced SVR and may respond well to norepinephrine and low-dose vasopressin as the first line of treatment, as these pressors allow for a low PVR/SVR ratio [36]. Dopamine and epinephrine should be avoided because of their tendency to cause tachycardia or myocardial oxygen demand in the face of potential RV ischemia [37]. Vasopressin at lower doses can actually decrease PVR by releasing NO from pulmonary vascular endothelium, but, at higher doses, it can cause RV ischemia [38]. Systemic hypotension with elevated PVR and right atrial pressure (RAP) suggests that high RV afterload and pulmonary vasodilator therapy may be required after hypotension is stabilized [11]. Inhaled prostacyclin analogs, NO, or iv sildenafil are rapid-acting [39,40].

### 4.6. Perioperative RV Dysfunction

If the above interventions with pulmonary vasodilators ± vasopressors fail, VA extracorporeal membrane oxygenation (ECMO) needs to be considered only as a bridge to urgent transplantation, especially if a reversible cause of RV failure like MI or PE has occurred [12]. Use of milrinone, especially in the inhaled form, can be considered, but only in conjunction with vasoconstrictors to avoid vasodilation and hypotension [12]. Data supporting this practice after NCS are scarce compared with those from cardiac surgical patients [41]. An intra-aortic balloon pump (IABP) is helpful in concomitant LV dysfunction where it helps improve RV flow by reducing the LA pressure. RV-assist devices are not recommended at this time.

## 5. Special Surgical Populations

### 5.1. Liver Transplantation

Porto-pulmonary hypertension (POPH) can be encountered in 5% of patients requiring liver transplantation, and this procedure has one of the highest rates of mortality (35%) in patients with PH. RHC is recommended for screening RVSP ≥ 40 mmHg. In up to 1/3 of patients awaiting liver transplant, the elevated MPAP may not be related to PVR, so therapies that can reduce MPAP to <35 mmHg (including diuretics) are encouraged during the preparation period [42,43]. A lack of randomized data, splenomegaly, and thrombocytopenia limit the usage of pulmonary vasodilators, specifically the prostacyclin analogues. A liver transplant should not be considered for MPAP > 35 mmHg or associated signs of RV failure. MPAP > 50 mmHg translates into a 100% postoperative mortality rate and is an absolute contraindication to a liver transplant and/or TIPS (MPAP ≥ 45 mmHg) [44].

### 5.2. Thoracic Surgery

Hypoxic pulmonary vasoconstriction is a major concern for patients undergoing thoracic surgery, especially when single lung ventilation (SLV) is required. The choice of video-assisted thoracoscopic surgery (VATS)/minimally invasive surgery over open thoracic surgery and inhaled pulmonary vasodilators can help mitigate some of these issues [45]. In patients with known or suspected PH, a lung biopsy for the diagnosis of parenchymal lung disease is best avoided. The worsening of PVR as well as arrhythmias can be expected with the re-expansion of the non-ventilated lung [46].

### 5.3. Orthopedic Surgery

A major concern with orthopedic surgery centers around pulmonary, fat, and/or cement embolization making an already high PVR worse. Elective hip and knee replacement surgeries carry high mortality rates and are not advised for patients with moderate-to-severe PH [47]. If PH is not moderately severe, then cementless joint replacements can be considered under epidural analgesia, with or without peripheral nerve blockade.

## 6. Future Directions

**Figure 1:**
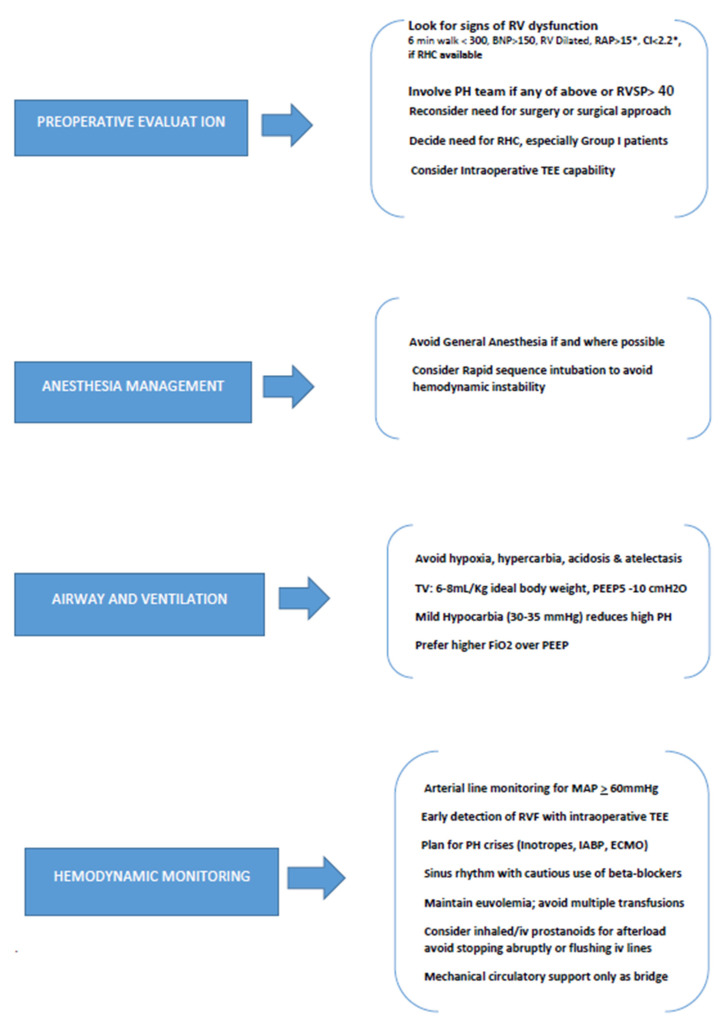
Perioperative evaluation and management of Pulmonary hypertension in Non-cardiac surgery. (Abbreviations: BNP: Brain natriuretic peptide, RAP: Right atrial pressure, CI: Cardiac index, RV: Right ventricle, PH: Pulmonary hypertension, RVSP: Right ventricular systolic pressure, RHC: Right heart catheterization, TEE: Trans-esophageal echocardiography, TV: Tidal volume, PEEP: Positive end expiratory pressure, MAP: Mean arterial pressure, RVF: Right ventricular failure, IABP: Intra-aortic balloon pump, ECMO: Extra-corporeal membrane oxygenation). * If prior Right heart catheterization results available.

Managing PH in the perioperative arena remains a significant challenge, as does the optimal recognition of PH before elective NCS. (Figure 1) No studies have been conducted comparing the outcomes of PH undiagnosed at the time of elective NCS with those of patients under treatment for PH. Although the risk of perioperative complications has been reported to be higher in group 1 PH, there are far more patients with PH relating to underlying heart, lung, metabolic, and other diseases [4,48]. These high-risk but otherwise minimally symptomatic patients may be considered for screening for occult PH prior to NCS. The role of echocardiography, CPET, risk stratification scores, or biomarkers in adequate screening needs to be investigated. One particularly high-risk group is elderly and or/obese patients with an ever-rising prevalence of HFpEF. Given that patients with PH are at risk for acute RVF, more attention needs to be directed to the detection of underlying chronic RV dysfunction/RVF, both preoperatively as well as postoperatively. Randomized trials should evaluate the effectiveness and safety of pulmonary vasodilators as “bridge therapy” in the perioperative period, as well as the eligibility of liver transplants in patients with POPH.

## Figures and Tables

**Table 1:** Things to avoid in the perioperative management of PH.

*1.*	*Patients with hemodynamic signs of RV failure (CVP > 15; low CO), hypoxia, or dyspnea at rest should not be taken for surgery.*
*2.*	*Patients with moderately severe PH should not undergo liver transplantation (for POPH) or joint replacement surgery.*
*3.*	*Avoid GA if and wherever possible.*
*4.*	*Avoid intubation if possible, especially in group 1 PH, by using PAP, inhaled pulmonary vasodilators and or, high-flow nasal cannula.*
*5.*	*Avoid volume overloading and hypotension.*
*6.*	*Do not flush the lines infusing pulmonary vasodilators.*
*7.*	*Do not abruptly stop pulmonary vasodilators.*
*8.*	*Mechanical circulatory support should only be used as a bridge therapy or where recovery is expected.*

**Table 2:** Modifiable risk in the perioperative management of PH.

*1.*	*Is the surgery necessary or alternative procedure/approach plausible?*
*2.*	*Can the patient be moved to a center of excellence in PH care?*
*3.*	*Decide the need for RHC before surgery, especially in patients with group 1 PH.*
*4.*	*Is the anesthesia plan modifiable?*
*5.*	*Neuraxial anesthesia should be utilized slowly or in combination with epidural anesthesia with arterial line monitoring.*
*6.*	*Decide about perioperative cardiopulmonary monitoring. Is intraoperative TEE with expertise in procedure available?*
*7.*	*Plan for perioperative pulmonary hypertensive crisis (e.g., Inotropes, IABP, ECMO) and discuss high-risk cases with transplant teams before going for Non-Cardiac Surgery.*
*8.*	*Use lower tidal volumes (6–8 mL/Kg of ideal body weight) and PEEP (5–10 cm H_2_O).*
*9.*	*Use PAP and supplemental oxygen wherever necessary.*
*10.*	*Maintain normal sinus rhythm during the intraoperative period; avoid beta-blockers and calcium channel blockers if RV failure is suspected.*
*11.*	*Maintain euvolemia and hemodynamics; avoid multiple blood products at the same time.*

## Data Availability

Not applicable.
